# Comparison of Glycated Hemoglobin Levels in Diabetic Psychiatric Patients, Before and During the Covid-19 Pandemic

**DOI:** 10.1192/j.eurpsy.2023.1684

**Published:** 2023-07-19

**Authors:** M. E. Anagnostaki, I. Anagnostaki, G. Papaspiropoulou

**Affiliations:** 1 Internal Medicine Dpt, Psychiatric Hospital of Attika, Athens, Greece

## Abstract

**Introduction:**

The current SARS Covid-19 pandemic has negatively affected primary care and health system services provided to chronically ill patients, such as patients with Diabetes Mellitus

**Objectives:**

The comparison of glycemic regulation as demonstrated by the levels of glycated hemoglobin HbA1c % in Psychiatric patients monitored at the Psychiatric Hospital of Attica before the pandemic (9/2018 -2/2020) and during its progression (3/2020-2/2022)

**Methods:**

The study was done retrospectively and included 543 diabetic patients who were examined in Outpatient Clinics during the aforementioned time intervals. HbA1c % levels were measured in peripheral blood and at least two measurements were averaged for each patient. The statistical method used to compare the mean value of HbA1c % was the paired t-test and the level of significance was p<0.05.

**Results:**

The mean of the mean values of HbA1c % before the pandemic was 7.22% while (sd 6.6-7.9) while during the pandemic period it was 8.56% (sd 7.1-9.9). The t score was calculated as 3.3 with a significance level of p=0.0165 (p<0.05).

**Conclusions:**

n the present study, a statistically significant increase in the HbA1c % of the Diabetic patients of the Psychiatric Hospital over the last two years is found, which indicates a worsening of the glycemic control of this particular group of patients. It is worth noting that 24 out of 543 (4.4%) missed a scheduled follow-up visit, while the greater variation in HbA1c % values during the second period indicates the varied way the pandemic affected the behavior of psychiatric patients

**Disclosure of Interest:**

None Declared

